# “Time is spine”: the importance of early intervention for traumatic spinal cord injury

**DOI:** 10.1038/s41393-020-0477-8

**Published:** 2020-05-11

**Authors:** Christopher S. Ahuja, Jetan H. Badhiwala, Michael G. Fehlings

**Affiliations:** 1 Department of Surgery, University of Toronto, Toronto, ON Canada; 2 Division of Genetics and Development, Krembil Research Institute, University Health Network, Toronto, ON Canada; 3 Spinal Program, Toronto Western Hospital, University Health Network, Toronto, ON Canada

**Keywords:** Health care, Cell death in the nervous system

Traumatic spinal cord injuries (SCI) have devastating lifelong sequelae for affected individuals, caregivers, and society. Modest improvements in sensorimotor function can significantly enhance quality of life and decrease costs of care. Early surgical decompression of the injured spinal cord is one of few available interventions that can potentially alter the long-term recovery trajectory for this devastating condition [1]. Early decompression has a sound pathophysiologic rationale, clinical evidence of efficacy, and international guideline recommendations as a treatment option.

## Pathophysiologic rationale for early surgical decompression

Traumatic injury to the spinal cord results in mechanical damage to neuroglial cell membranes, the extracellular matrix architecture, and the sensitive microvasculature. This initiates a secondary injury cascade from the moment of injury which causes additional damage and neurologic dysfunction. Within minutes, hemorrhage and blood-spinal-cord barrier disruption allow a rapid influx of peripheral inflammatory cells (e.g. polymorphonuclear leukocytes and macrophages) and cytokines (e.g. interleukin-1β, tumor necrosis factor-α, etc.) to the site of injury. This, combined with significant ionic dysregulation and the release of byproducts of cellular necrosis (e.g. DNA, ATP, neurotransmitters), initiates proapoptotic signaling in nearby cells. As phagocytes clear debris, they generate free radicals which can generate additional cellular injury. This is compounded by the compromised microvasculature which can create a lesional and perilesional region of ischemia. As the injury evolves over hours, increasing edema and ongoing compression of the cord cyclically add to neuroglial cell death [2]. Surgical decompression aims to interrupt this progression by relieving pressure within the cord parenchyma to partially restore microvascular blood flow, reduce ischemia, and remove mechanical stretch/compression of neuroglial cell membranes. To this point, there is robust basic science data from rodent models of SCI to indicate that the degree of histological tissue destruction is mitigated, and neurobehavioral outcomes improved, when the duration of compression is minimized [3, 4].

## The evidence for early surgical decompression in acute spinal cord injury

The bulk of clinical evidence favors a 24-h threshold in defining “early” surgical decompression [5–8]. The results of the Surgical Timing in Acute Spinal Cord Injury Study (STASCIS) were published in 2012 [9]. This was a multi-center, nonrandomized, prospective cohort study that enrolled 313 adults (18–80 years) with cervical SCI from six North American centers. Of these, 182 patients underwent early surgical decompression (<24 h), whereas 131 received late surgery (≥24 h). The primary endpoint was change in American Spinal Injury Association Impairment Scale (AIS) grade at 6 months post-injury. A significantly greater proportion of patients who underwent early surgery (19.8%) demonstrated a 2-or-more grade improvement in AIS at 6 months compared with those who underwent late surgical intervention (8.8%). A positive effect of early surgical decompression on ≥2 grade improvement in AIS persisted after adjustment for pre-operative neurological status and steroid administration (OR 2.83, 95% CI 1.10–7.28). Complication rates were comparable between early (24.2%) and late (30.5%) surgery groups. Since publication of STASCIS, there has been a growing body of evidence supporting the safety and efficacy of early surgical decompression, undertaken within 24 h of injury, for acute traumatic SCI [10]. Although a 24-h threshold is frequently defined, it is important to acknowledge that the biological rationale for early surgery in mitigating secondary injury would favor minimizing the time from injury to decompression as much as possible. To that end, there is emerging evidence to indicate that even shorter time thresholds–for example, 12 or 8 h–may be associated with superior neurological recovery in SCI patients [11, 12]. However, these studies included very small patient cohorts and further validation of these findings, ideally in a prospective fashion, is necessary.

## The special case of central cord syndrome

With the continued aging of the global population, central cord syndrome (CCS) is soon expected to become the most common form of acute SCI. These injuries classically arise from a hyperextension mechanism in an older person with pre-existing cervical spondylosis; the clinical hallmark is greater hand and arm than lower limb weakness [10]. The natural history of CCS is thought to entail good recovery, and historically, surgical intervention has been disfavored. However, the rationale for early surgical decompression may perhaps be strongest in the context of CCS. Considering that CCS classically results from lower energy mechanisms (e.g., falls) that are often insufficient to produce fracture and/or dislocation of the spinal column, the primary injury to the spinal cord is likely proportionally smaller, for example, as compared with SCI resulting from cervical facet dislocation. On the other hand, secondary injury to an edematous spinal cord from ongoing compression from a stenotic central canal may play a dominant role in CCS, meaning that there may be a greater window of opportunity to afford neuroprotection to the spinal cord with early decompression. Nonetheless, there remains a paucity of contemporary high-quality data relating to the efficacy of early surgical decompression in the setting of CCS [13]. This remains a critical knowledge gap today, and indeed, a public health priority, considering the aging population and growing incidence of these injuries.

## Barriers to early surgery

The acute care of individuals with spinal cord injury is complex, highly-specialized, and often associated with polytrauma. Barriers to early surgical decompression may include transport logistics from the field, access to a hospital and surgeon providing spine care, operating room availability, and intensive care resources [14]. Additional delays can occur in multiple diagnostic steps such as initial clinical recognition of SCI, as well as obtaining and interpreting advanced imaging. Studies from North America and Europe have suggested that only 20–50% of patients with acute SCI are transferred to a spine-care center within 24 h of injury where decompression becomes an option [10, 15]. Therefore, the majority of patients arrive at these centers outside of the early decompression window where timely intervention could translate into potential long-term functional improvement [10].

## Clinical practice guideline recommendations on the timing of surgical decompression for acute spinal cord injury

The most recent clinical guidelines for the management of acute SCI were published in 2017. These were sponsored by AO Spine North America, AO Spine International, and the American Association of Neurological Surgeons/Congress of Neurological Surgeons Joint Section on Neurotrauma and Critical Care. With regard to the timing of surgical decompression, these guidelines provide two weak recommendations based on low-quality evidence: (1) “We suggest that early surgery be offered as an option for adult acute SCI patients regardless of level”; and (2) “We suggest that early surgery (≤24 h after injury) be considered as a treatment option in adult patients with traumatic CCS” [16].

## Challenges in knowledge translation

While literature evidence is strong to support the “Time is Spine” concept, adoption has not been ubiquitous. This suggests a greater need to translate the knowledge gained from the above studies and to disseminate current practice guidelines widely to improve patient care. It is also important to recognize that achieving early decompression in developing countries can be challenging where rates of neurotrauma are high but infrastructure may be limited. This also represents a significant opportunity to continue improving worldwide spine care.
